# Duodenal Leiomyosarcoma Mimicking a Pancreatic Pseudocyst

**DOI:** 10.1155/1994/71873

**Published:** 1994

**Authors:** C. Sperti, C. Pasquali, F. Di Prima, R. Baffa, S. Pedrazzoli

**Affiliations:** 1 Department of Surgery University of Padua Padova Italy; 2 Department of Radiology Cittadella Hospital City Cittadella, Padova Italy; 3 Department of Pathology Cittadella Hospital City Cittadella, Padova Italy; 4 Semeiotica Chirurgica University of Padua Via Facciolati 71 Padova 35127 Italy

## Abstract

A case of duodenal leiomyosarcoma presenting as a cystic mass is reported. Amylase, tumour markers levels in the cyst fluid and radiological findings suggested an inflammatory pancreatic pseudocyst. Exploratory laparotomy and frozen section examination showed a smooth muscle tumour of the duodenum. Pancreatoduodenectomy with pylorus-preser vation was performed and the patient remained symptom-free at 8 months follow-up.


*HPB Surgery*, 1994, Vol. 8, pp. 49–52
Reprints available directly from the publisher
Photocopying permitted by license only

© 1994 Harwood Academic Publishers GmbH
Printed in Malaysia

### Duodenal Leiomyosarcoma Mimicking a Pancreatic Pseudocyst

C. SPERTI, C. PASQUALI, F. DI PRIMA*, R. BAFFA† and S. PEDRAZZOLI

Department of Surgery, University of Padua, Padova, Italy,
Department of Radiology* and Pathology†, Cittadella Hospital City, Cittadella, Padova, Italy.

A case of duodenal leiomyosarcoma presenting as a cystic mass is reported. Amylase, tumour markers levels in the cyst fluid and radiological findings suggested an inflammatory pancreatic pseudocyst. Exploratory laparotomy and frozen section examination showed a smooth muscle tumour of the duodenum. Pancreatoduodenectomy with pylorus-preser vation was performed and the patient remained symptom-free at 8 months follow-up.

KEY WORDS: Leiomyosarcoma duodenal tumours pancreatic pseudocyst cystic neoplasms

### INTRODUCTION

Leiomyosarcoma of the duodenum is uncommon and accounts for approximately 10% of malignant duodenal tumours[1]. The CT appearance of gastrointestinal leiomyosarcoma may be that of a cystic mass, due to cavitation and necrosis of the tumour[2].

This report describes a patient whose cystic duodenal leiomyosarcoma clinically mimicked an inflammatory pancreatic pseudocyst.

### CASE REPORT

A 65 year-old, obese, woman presented in December 1991 with epigastric pain, radiating to the back, and intermittent fever of 4-days duration. The past medical history revealed epigastric discomfort for 5 years; her alcohol ingestion was estimated as 100 g/day for 20-years and she denied abdominal trauma. Routine laboratory tests showed high alkaline phosphatase levels (409 IU/L; n.v. 98–279), slight increase of serum transaminases, amylase (138 IU/L; n.v. < 90 IU/L) and lipase (1139 IU/L; n.v. < 200 IU/L). Serum CA 19–9 and CEA levels were 21 U/ml (n.v. < 37 U/ml) and 1.5 ng/ml (n.v. < 10 ng/ml) respectively. Ultrasound showed multiple small stones in the gallbladder, no dilatation of the biliary tree and a 7-cm hypoechoic mass on the right of the abdomen. The pancreas was not clearly seen. An upper G.I. endoscopy was performed and revealed only mild antral gastritis and duodenitis. Computed tomography demonstrated a 9 cm low density mass (Figure 1), with an air-fluid level (Figure 2), in the region of the pancreatic head, which was compressing the inferior vena cava. No para-aortic lymph node enlargement was seen. A diagnostic aspiration of the cyst was performed and about 200 ml of a cloudy, brownish fluid was obtained. The amylase content in the fluid was 26,400 IU/L and the lipase was 61 IU/L. CA 19–9 and CEA levels in the cystic fluid were respectively 42 U/ml and 81 ng/ml; the Gram stain revealed a significant number of gram-negative rods. Percutaneous fine-needle aspiration cytology showed only necrotic material. Decompression of the cyst was confirmed by CT and drainage was established via a percutaneous catheter. Contrast injected into the cyst showed no communication with the pancreatic duct or intestinal lumen. Thereafter,

*Address correspondence to*: S. Pedrazzoli, Professor of Surgery, Semeiotica Chirurgica. University of Padua, Via Facciolati 71, 35127 Padova, Italy.

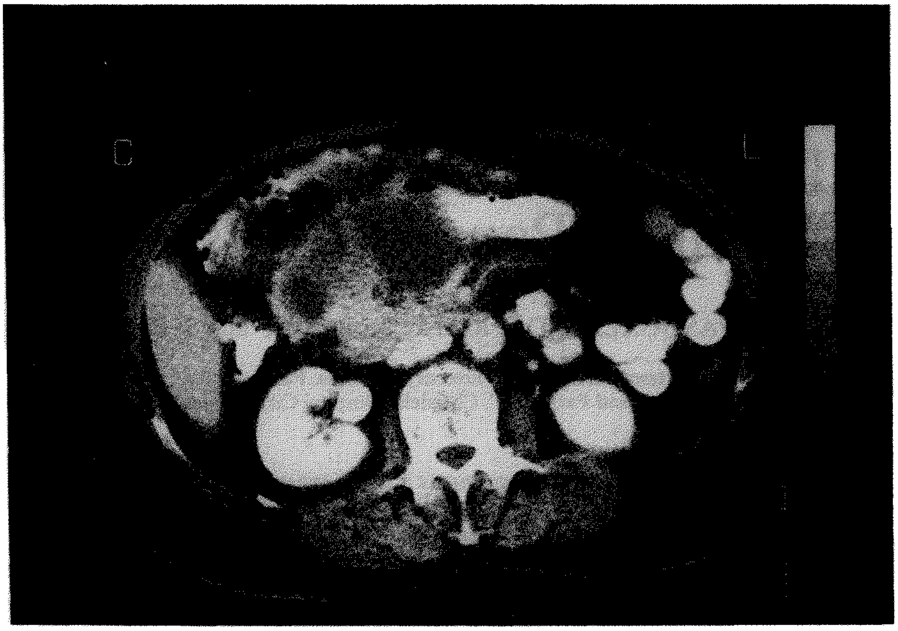

**Figure 1** Computed tomography of the abdomen showing a low density mass in the region of pancreatic head.

about 80 ml of purulent fluid drained daily; and a clinical diagnosis of infected pancreatic pseudocyst was made. After 8 days the clinical picture did not improve and the cyst enlarged again on CT examination. An exploratory laparotomy was then performed: a multiloculated cystic mass, arising from the second part of the duodenum, was demonstrated. Biopsy of the cyst wall submitted for frozen section examination revealed a smooth muscle tumor of the duodenum. Exploration of the abdominal cavity showed no metastases, thus a pancreaticoduodenectomy with pylorus-preservation was performed. Macroscopic examination of the

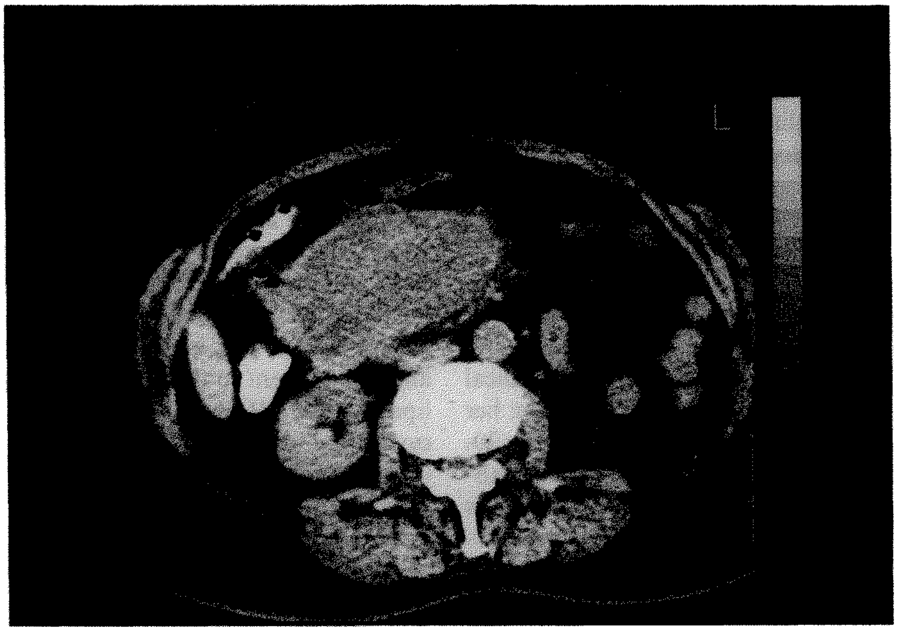

**Figure 2** Selected cut of CT abdominal scan showing a large mass, with air-fluid level.

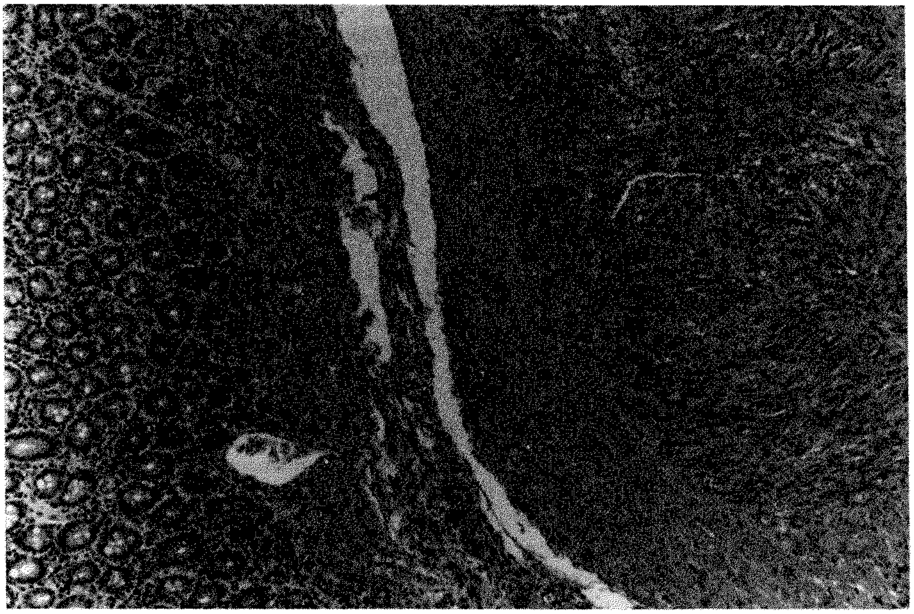

**Figure 3** A well-differentiated leiomyosarcoma of the duodenum: infiltrating the muscolaris mucosae: typical fascicular pattern with tumor bundles intersecting at right angles (HPE, × 100).

operative specimen disclosed a 10-cm multiloculated rubbery tumour containing hemorrhagic fluid. Microscopic examination showed a well differentiated duodenal leiomyosarcoma (Figure 3), infiltrating the periduodenal tissue. Between one and three mitotic figures were visualized per 10 high power fields. There was no metastatic spread in the 21 lymph nodes examined.

### DISCUSSION

A correct preoperative diagnosis of cystic masses, arising from the pancreatic or peripancreatic region, is important for appropriate treatment of these lesions. Warshaw and Rutledge[3] proposed multiple guidelines to differentiate pseudocysts from cystic tumors. Unfortunately, a variety of cystic neoplasms or other lesions mimicking pancreatic pseudocysts in their presentation, have been reported[4–8].

Our patient presented with a cystic mass in the region of the pancreatic head, without a history of pancreatitis or abdominal trauma. However, serum amylase and lipase levels were mildly elevated and the cyst fluid amylase level was high, suggesting pancreatic juice in the contents. These features are commonly seen in pancreatic pseudocysts, but cases of cystadenoma[5] and cystadenocarcinomas of the pancreas[6,8] with high amylase concentrations in the cyst fluid have been reported. Serum CA 19–9 and CEA levels were within the normal range, and CA 19–9 in the cystic fluid was at the upper limit of normal. These findings are indicative of a benign lesion. The CEA level in the aspirated fluid was high, but abnormal concentrations of CEA in pancreatic pseudocysts have been reported[9]. Tatsuta and coll.[9] showed that determination of tumour marker levels in cyst aspirate may be valuable in differentiating benign from malignant disease. On the other hand CA 19–9 or CEA levels in mesenchymal tumours are not reported. Fine-needle aspiration with cytologic examination may help in the differential diagnosis. However, in our patient it showed only necrotic tissue. A definitive diagnosis was made only by an intraoperative biopsy of the cyst wall with frozen section examination.

US and CT findings did not suggest a cystic tumour, showing a unilocular cyst. without septa, which is the appearance of a pancreatic pseudocyst. The CT-finding of air/or air-fluid level can be seen frequently in gastrointestinal leiomyosarcomas[2]; however, in our case the air-fluid level might be explained by infection in the cyst. On the other hand a communication with the intestinal lumen had been shown neither by contrast cystography nor by gastroduodenoscopy. In fact, leiomyosarcomas grow in an extraluminal direction in 63–75% of cases[10]; in our case the tumour grew extraluminally, reaching a large size.

Prognosis of duodenal leiomyosarcoma varies in the literature; the 5-year survival rate is about 50% or less[11], but long-term survival after surgery has been reported for tumours involving adjacent organs, suggesting an aggressive surgical approach whenever possible[12,13].

Although rare, duodenal leiomyosarcoma may be included in the differential diagnosis of peripancreatic cystic lesions, as shown by our case.

In recent years percutaneous aspiration of pancreatic pseudocysts, has become accepted as a safe and attractive alternative to a surgical strategy. In our case the suspicion of a pancreatic pseudocyst was made on the basis of cholelithiasis and the high amylase content in the cyst fluid. Many authors have reported a high success rate (90%) for percutaneous catheter drainage (P.C.D.) in the management of pancreatic pseudocysts[14], even in large fluid collections[15] P.C.D. is generally contraindicated in the presence of ductal stictures on ERCP, a high density appearance on CT scan and infection[16]. P.C.D., of course, should be avoided when a cystic tumour is suspected.

In our patient the high CEA level in the cyst fluid and the negative clinical history made the differential diagnosis more difficult. On the other hand, the CT appearance of necrotic material in the cyst, the infection of the cyst, the persistence of clinical symptoms and early reaccumulation of a cystic collection, were the relevant factors that made surgery the advisable option.

Thus we recommend greater caution in carrying out percutaneous drainage of these lesions, we suggest exploratory laparotomy whenever clinical and radiological findings do not provide a certain diagnosis.

### REFERENCES

1. Wilson, J. M., Melvin, D. B., Gray, J. F. and Thorbjarnorsson, B. (1974) Primary malignancies of the small bowel. *Annals of Surgery*, **180**, 175–179.
2. Kanematsu, M., Imaeda, T., Iinuma, G., Mori, S., Yamawaki, Y., Doi, H., Takao, H. and Shimowaka, K. (1991) Leiomyosarcoma of the duodenum. *Gastrointestinal Radiology*, **16**, 109–112.
3. Warshaw, A. L. and Rutledge, P. L. (1987) Cystic tumors mistaken for pancreatic pseudocysts. *Annals of Surgery*, **205**, 393–398.
4. Childs, C. G., Korsten, M. A., Choi, H. H., Schwarz, R. and Fisse, R. D. (1985) Pancreatic choriocarcinoma presenting as inflammatory pseudocyst. *Gastroenterology*, **89**, 426–431.
5. Sachs, J. R., Deren, J. J., Sohn, M. and Nusbaum, M. (1989) Mucinous cystadenoma: pitfalls of differential diagnosis. *American Journal of Gastroenterology*, **84**, 811–816.
6. Isaacs, P., Pinder, C., Jourdan, M., Filipe, I. and Sladen, G. (1986) Therapeutic aspiration of pseudocysts: a cautionary tale of the pancreas. *American Journal of Gastroenterology*, **81**, 1087–1090.
7. Conter, R. L., Converse, J. O., Mc Garrity, T. J. and Koch, K. L. (1990) Afferent loop obstruction presenting as acute pancreatitis and pseudocyst: case reports and review of the literature. *Surgery*, **108**, 22–27.
8. Railey, D. J., Barkin, J. S. and Levi, J. (1991) Aspiration of cystadenocarcinoma mimicking pancreatic pseudocyst. *Pancreas*, **6**, 491–494.
9. Tatsuta, M., Iishi, H., Ichii, M., Noguchi, S., Yamamoto, R., Yamamura, H. and Okuda, S. (1986) Values of carcinoembrionic antigen, elastase 1 and carbohydrate antigen determinant in aspirated pancreatic cystic fluid in the diagnosis of cysts of the pancreas. *Cancer*, **57**, 1836–1839.
10. McBrien, M. L. and Jarrett, P. E. M. (1971) Leiomyosarcoma of the duodenum. *British Journal of Surgery*, **58**, 658–689.
11. He, L. J., Wang, B. S. and Chen, C. C. (1988) Smooth muscle tumors of the digestive tract: report of 160 cases. *British Journal of Surgery*, **75**, 184–186.
12. Watanabe, A., Korenaga, D., Baba, H., Tsujitani, S., Kakeji, Y., Mori, M. and Sugimachi, K. (1990) Long survival of a woman with huge leiomyosarcoma of the duodenum after pancreaticoduodenectomy and transverse colectomy: a case report. *Surgery*, **108**, 110–113.
13. Perdrazzoli, S., Sperti, C., Militello, C., Chiappetta, A., Pomerri, F. and Petrin, P. (1984) Duodenal leiomyosarcoma and its multiple recurrences: good surgical results after three years of follow-up. *Italian Journal of Surgical Sciences*, **14**, 321–326.
14. Von Sonnenberg, E., Vittich, G. R., Casola, G., Branningan, T. C., Kernel, F., Stabile, B. E., Varney, R. R. and Christensen, R. R. (1989) Percutaneous drainage of infected and non-infected pancreatic pseudocysts: Experience in 101 cases. *Radiology*, **170**, 757–761.
15. Adams, D. B. and Anderson, M. C. (1992) Changing concepts in the surgical management of pancreatic pseudocysts. *American Surgeon*, **58**, 173–180.
16. D'Egidio, A. and Schein, M. (1991) Percutaneous drainage of pancreatic pseudocysts: a prospective study. *World Journal of Surgery*, **16**, 141–146.

